# Prostate-specific RNA aptamer: promising nucleic acid antibody-like cancer detection

**DOI:** 10.1038/srep12090

**Published:** 2015-07-15

**Authors:** Karina Marangoni, Adriana F. Neves, Rafael M. Rocha, Paulo R. Faria, Patrícia T. Alves, Aline G. Souza, Patrícia T. Fujimura, Fabiana A. A. Santos, Thaise G. Araújo, Laura S. Ward, Luiz R. Goulart

**Affiliations:** 1 Laboratory of Cancer Molecular Genetics, Faculty of Medical Sciences, State University of Campinas, SP, Brazil; 2 Laboratory of Nanobiotechnology, Institute of Genetics and Biochemistry, Federal University of Uberlândia, MG, Brazil; 3 Laboratory of Molecular Biology, Institute of Biotechnology, Federal University of Goiás, Catalão/GO, Brazil; 4 AC Camargo Cancer Hospital, SP, Brazil; 5 Laboratory of Histology, Institute of Biomedical Sciences, Federal University of Uberlândia, MG, Brazil; 6 University of California-Davis, Dept. of Medical Microbiology and Immunology, Davis/CA, USA

## Abstract

We described the selection of a novel nucleic acid antibody-like prostate cancer (PCa) that specifically binds to the single-stranded DNA molecule from a 277-nt fragment that may have been partially paired and bound to the *PCA3* RNA conformational structure. *PCA3*-277 aptamer ligands were obtained, and the best binding molecule, named CG3, was synthesized for validation. Aiming to prove its diagnostic utility, we used an apta-qPCR assay with CG3-aptamer conjugated to magnetic beads to capture *PCA3* transcripts, which were amplified 97-fold and 7-fold higher than conventional qPCR in blood and tissue, respectively. Histopathologic analysis of 161 prostate biopsies arranged in a TMA and marked with biotin-labeled CG3-aptamer showed moderate staining in both cytoplasm and nucleus of PCa samples; in contrast, benign prostatic hyperplasia (BPH) samples presented strong nuclear staining (78% of the cases). No staining was observed in stromal cells. In addition, using an apta-qPCR, we demonstrated that CG3-aptamer specifically recognizes the conformational *PCA3*-277 molecule and at least three other transcript variants, indicating that long non-coding RNA (lncRNA) is processed after transcription. We suggest that CG3-aptamer may be a useful PCa diagnostic tool. In addition, this molecule may be used in drug design and drug delivery for PCa therapy.

Aptamers are short nucleic acids that fold into a well-defined three-dimensional structure enabling their interaction with molecules with high affinity and specificity^1^. They are considered a novel promising class of compounds with diagnostic and therapeutic potentials that may substitute antibodies, which larger molecules, greater immunogenicity and peptidase susceptibility limit their pharmacological value^2^.

SELEX (Systematic Evolution of Ligands by EXponentional enrichment) is the technology used for *in vitro* selection of aptamers^3^,^4^, evolving ligands against single molecules to complex target mixtures, or even whole organisms. Other targets, such as RNA, have only been used to improve the understanding of RNA–RNA interactions^5^. The most competitive aptamers can participate in complex regulatory networks, not only by Watson–Crick interactions with other RNA, but also by forming discrete secondary structures that are able to bind DNA^6^, RNA or protein targets^7^.

Aptamer specificity and affinity targeting RNA secondary structure is higher than that of complementary oligonucleotides^8^. Interesting effects of aptamers, either degrading RNA or inhibiting RNA functions have been reported, suggesting their potential role as therapeutic agents targeting long non-coding RNAs (lncRNAs). In addition, some aptamers may be used to modulate viral gene expression by interacting with viral RNAs^9^.

Non-coding RNAs (ncRNAs) fall in a broad range of regulatory RNA molecules such as ribozymes, antisense, small interfering RNAs or aptamers that are either naturally found in several cell types or artificially designed to target genes and control their expression^10^. Most lncRNAs, yet to be thoroughly characterized, exhibit significant cell type-specific expression, subcellular compartments localization, and are associated with human diseases^11^. Among the many putative regulatory paradigms of these molecules, they may serve as structural RNAs that will be part of the formation of RNA–protein or RNA–RNA complexes^12^, which may be critical in regulating the activity or localization of proteins, or serve as organizational frameworks of subcellular structures.

As a proof-of-concept for structural ncRNAs, we investigated the lncRNA prostate cancer antigen 3 (*PCA3*). This transcript is overexpressed in more than 95% of primary prostate cancer (PCa) tissue specimens^13^ and is the first PCa specific biomarker described^14^, being involved in PCa cell survival and modulation of androgen receptor (AR) signaling^15^. An *in silico* analysis generated a remarkable tertiary structure of *PCA3* transcript with a significant free energy, showing many hairpins and loops due to extensive base pairing within the molecule. Our hypothesis was that this conformational structure could be functional and required either for editing or processing, eventually controlling other genes, as previously described^15^. It is interesting to point out that functional RNAs have a more stable secondary structure than expected by chance, since most known functional RNAs depend on a defined secondary structure^16^.

High affinity aptamer ligands to *PCA3* transcript can also serve as templates for real-time PCR (qPCR), possibly to improving PCa diagnosis. The assay format coined as real-time apta-PCR (apta-qPCR) is an extension of immuno-PCR^17^, where the DNA-labeled antibody is replaced by a non-labeled aptamer, which, in turn, acts both as a reporter and as biorecognition molecule. Whilst offering great sensitivity, immuno-PCR technique suffers from some important drawbacks, such as difficulties in labeling the antibody with nucleic acids. Furthermore, this binding lacks precision due to an uneven number of oligos *per* antibody, resulting in high error rates^18^. Apta-qPCR overcomes the aforementioned limitations, further highlighting the exquisite advantages of aptamers.

Our investigation describes the selection of a novel nucleic acid antibody-like for the *PCA3* detection, an RNA aptamer that specifically binds to the single-stranded DNA molecule from a 277-nt fragment that may have been partially paired and bound to the *PCA3* RNA conformational structure. We selected six ligands to the lncRNA conformational structure with high affinity to the molecule, which were further characterized and applied in several assay formats with significant implication for the PCa diagnosis. Finally, CG3-aptamer applicability was validated using a tissue microarray, and also by magnetic capture followed by qPCR (apta-PCR) in both tissue and peripheral blood, under physiological conditions, without controlling hybridization parameters.

## Results

### Aptamers that recognize RNA conformational structures

**Figure 1:**
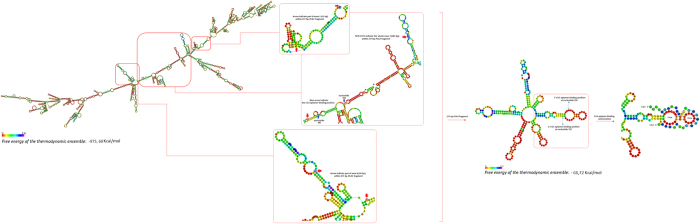
Structure prediction analyses for *PCA3* transcript. Insets (red squares) indicate the regions that encompass the 277-nucleotide transcript within *PCA3.* Conformational changes of *PCA3* target site and its putative interaction with CG3-aptamer, according to *StarMir* prediction based-pairing.

Our *in silico* analysis of the *PCA3* transcript generated a remarkable tertiary structure with a highly significant free energy (ΔG > −975.60 kcal/mol), showing an extensive base pairing within the molecule that led to a very constrained structure with many hairpins and loops (Fig. 1). We successfully generated a genomic library amplified by PCR after eight rounds of *in vitro* selection against the single-stranded DNA molecule from a 277-nt fragment that may have partially been paired and bound to the *PCA3* RNA conformational structure. Genomic SELEX allowed the identification of RNA fragments that might reflect natural interactions with the chosen target.

The selection steps were continuously performed at 37 °C. The PCR product resulted from 1^st^, 3^th^ and 8^th^ rounds were cloned and sequenced. Clone sequences were aligned and consensus motifs were identified using the *Clustal software package* of the GCG suite of molecular biology programs.

**Figure 2:**
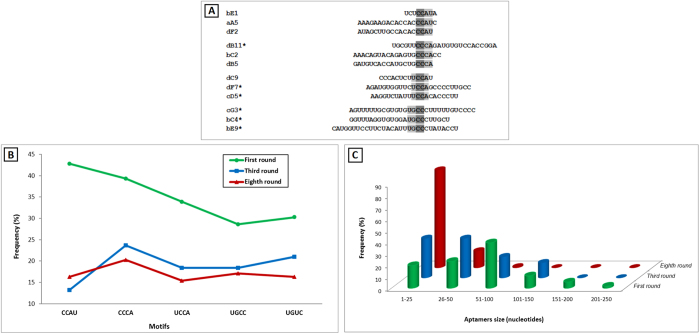
(**A**) Sequence alignments of motifs from the 8^th^ selection round. Five motifs were identified (CCAU, CCCA, UCCA, UGCC and UGUC) within the best six aptamers (*). Frequencies of selected motifs (**B**) and sequence sizes (**C**) for 10^15^ random-sequence pools, across the 1^st^, 3^rd^ and 8^th^ selection cycles.

The search for motifs is a strategy to establish a convergent evolution of RNA molecules during selection in order to identify the most significant and repetitive short nucleotide sequences. Sequences were classified according to the presence of five different motifs identified (CCAU, CCCA, UCCA, UGCC and UGUC) during the 1^st^, 3^th^ and 8^th^ selection rounds. Some sequences presented more than one motif, thus they appeared in more than one family classification. Sequence alignment demonstrated that the four motifs (CCAU, CCCA, UCCA and UGCC) presented one common “CC” dinucleotide repeat (Fig. 2A). Heterogeneity of motifs among SELEX rounds were observed, but variability within each round was low (Fig. 2B). The CCCA, UGCC and UGUC motifs were the most frequent in the 3^th^ and 8^th^ round, and screening led us to the selection of 88 out of 123 valid sequences from the last round.

Sequences smaller than 21 nucleotides were excluded from the analysis. Our results showed that the average sequence length dropped from 66 bases to an average 47 bases upon initial transcription and reverse transcription in the first selection round, and steadily declined in remainder selection rounds, achieving approximately 21 bases at the 8^th^ round (Fig. 2C). More than half of the sequences were excluded in the trimming and alignment processes by *in silico* analyses (48 out of 88 clones).

The free energy minimization between the secondary structure target (*PCA3*) and aptamers was predicted by *RNAhybrid*, as an extension of the classical RNA secondary structure prediction algorithm^19^ for two sequences. The established algorithm computes the optimal folding and a set of suboptimal foldings^20^. To identify potential sites with a stable hybrid, we used a threshold of ΔG_hybrid_ ≤ −18 kcal/mol as a measure of hybrid stability, and only six sequences (out of 40) were specific to *PCA3-277*. It is interesting to observe that the predicted hybridization regions between aptamers and *PCA3-277* presented two conserved binding domains in *PCA3-277,* located at the positions 2 to 26 and from 186 to 218 bases, corresponding to exons 1 and 3, respectively (Table 1).

The structure predictions exhibited two types of conserved domains; one that formed structural scaffolds, and another that was frequently represented by single-stranded or non-canonical base-pairing that directly interacted with the target molecule^20^. Results presented in Table 1 show that the CG3-aptamer presented more bases paired with a *PCA3-277* loop, with a binding energy of ΔG_hybrid_ = −19.8 kcal/mol (Fig. 1).

Finally, to demonstrate whether *PCA3-277* is in fact a linear or conformational structure, we performed binding assays with denatured and native RNA molecules by dot-blot (solid-phase), immunohistochemistry, and by magnetic capture in solution. Dot-blot assays were not effective due to baking and UV cross-linking procedures, which may have disturbed the native structure. The other assays, performed under physiological conditions, have successfully resulted in positive detection only of the native folded structure. The binding of aptamers (CG3 and BC4) to the denatured *PCA3* RNA, heated at 97 °C for 5 min, could not capture the molecule, resulting in negative results after PCR amplification assays. If *PCA3* molecules had been bound to the aptamers, they would have been eluted by the alkaline treatment prior to PCR amplification, and results would be positive.

### Apta-qPCR assay for PCa diagnosis

**Figure 3:**
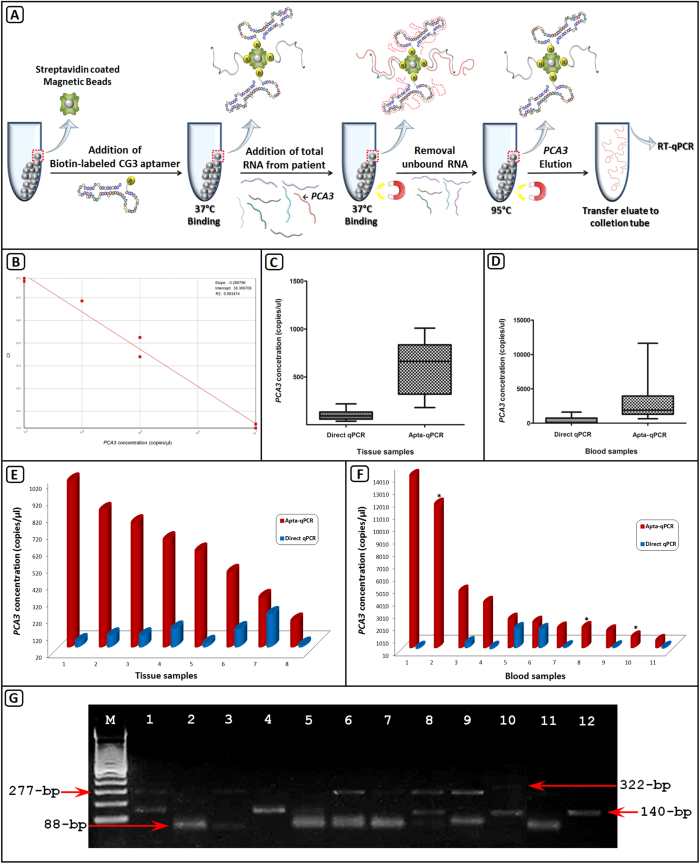
(**A**) Schematic overview of the apta-qPCR approach. (**B**) Standard curve constructed using 10^7^ to 10^2^ copies/μL of *PCA3*. Data were obtained from six independent experiments with duplicate samples at four time points, and show high PCR reproducibility and efficiency. Comparisons of direct qPCR and apta-qPCR in tissue (**C**) and blood (**D**) samples. *PCA3* levels (copies/μL) calculated for tissue (**E**) and blood (**F**) samples. (**G**) Agarose gel electrophoresis analysis of *PCA3* amplicons. Red arrows indicate *PCA3* variant sizes. Lanes 1 and 4 = direct qPCR for tissue samples; lanes 8, 10 and 12 = direct qPCR for blood samples; lanes 2, 5 and 11 = apta-qPCR for tissue samples; lanes 3, 6, 7 and 9 = apta-qPCR for blood samples. M = 100-bp ladder.

We hypothesized that PCa diagnosis could be improved using CG3-aptamer as a capturing molecule prior to a qPCR assay (Fig. 3A). Hence, we developed an apta-qPCR assay to detect *PCA3* transcripts by using magnetic capture with CG3-aptamer.

Herein, the selected CG3-aptamer was evaluated for its potential clinical utility using the apta-qPCR assay, combining the sensitivity of nucleic acid amplification and the selectivity of aptamers^21^. Biotin-labeled CG3-aptamer, immobilized onto streptavidin-coated magnetic beads, was used to capture the *PCA3* conformational RNA molecule, and its ability to bind *PCA3-277* asymmetric PCR fragments was tested in serial dilutions of the transcript. The binding efficiency of aptamer was evaluated based on the amount of *PCA3-277* recovered by elution and quantified by qPCR. Our data demonstrated that the reaction efficiency and linearity was consistent. The calibration curve presented 100% efficiency (Fig. 3B), and the limit of detection of the *PCA3* assay was <10^2^ copies *per* mL. The amount of *PCA3* recovered by apta-qPCR was directly proportional to the amount of *PCA3* initially incubated with biotin-labeled CG3-aptamer, which is an indicator of high affinity and specificity of the aptamer. Theoretically, each magnetic bead presents three streptavidin binding sites, allowing three molecules of biotin-labeled CG3-aptamer binding (Pierce™, Thermo Scientific technical information).

To further validate the usefulness of the apta-qPCR assay, specific analyses of *PCA3* expression were performed in 8 human tissues and 11 peripheral blood samples with a wide linear dynamic range of magnitude. After magnetic capture of *PCA3* transcript by biotin-labeled CG3-aptamer, the bound *PCA3* was heat-eluted and quantified by qPCR. The limit of quantification (LOQ) was determined by fluorescence intensity, according to the concentration of mRNA in the PCR reaction, and comparisons between apta-qPCR and direct qPCR in both tissues and blood were performed. All tissue samples were *PCA3* positive for both apta-qPCR and direct qPCR assays. We have achieved an LOQ 7-fold higher for apta-qPCR compared with direct qPCR (p < 0.05) (Fig. 3C). The blood samples that were *PCA3* negative for direct qPCR analysis (3 out of 11) were successfully detected by the apta-qPCR (sensitivity = 100%). The LOQ was 97-fold higher for apta-qPCR compared with direct qPCR (p < 0.05) (Fig. 3D), suggesting that aptamer-coupled to magnetic beads is able to capture more molecules than by direct qPCR.

Therefore, the apta-qPCR may significantly improve the sensitivity of PCa diagnosis. BPH patients (n = 2) and young healthy individuals used as negative controls (n = 2) were consistently negative in all PCR replicates. In addition, the complementary nature of specific sequences of different sizes in the same region of *PCA3* (277-bp) have been tried, and they only worked as primers, under denatured conditions, as observed in conventional PCR reactions. None of the sequences could detect the molecule under native conditions. Importantly, other aptamers generated in the same selection have been tested, but with lower sensitivity, and specifically BC4 (Supplementary Table 1) were able to capture only half of *PCA3* molecules in comparison with CG3.

The limit of detection has been reported elsewhere^22^ to be three molecules *per* PCR reaction, and reliable quantification can be performed above the quantification limit of 800 *PCA3* mRNA copies *per* milliliter of blood.

To further confirm whether the complete *PCA3-277* fragment was fully captured and amplified, all amplicons obtained by direct qPCR and also by agarose gel electrophoresis analysis were compared with those from apta-qPCR reactions for both tissues and blood (Fig. 3E,F, respectively), (Fig. 3G). Three specific fragments were observed and matched the variant sizes and sequences reported by our group (unpublished work); 88-bp (gi:87245044), 140-bp (gi:87245043), and 322-bp (gi:87245042). The 88-bp fragment was the most frequent amplicon in the apta-qPCR (100% in the blood), but rarely amplified by direct qPCR. Therefore, the fluorescence intensity obtained in the real-time qPCR corresponded to the sum of fluorescence signals of any amplified fragment in each reaction. *PCA3* variants observed may be an indication that lncRNA is processed after transcription.

### Aptamers as histological probe for PCa detection

**Figure 4:**
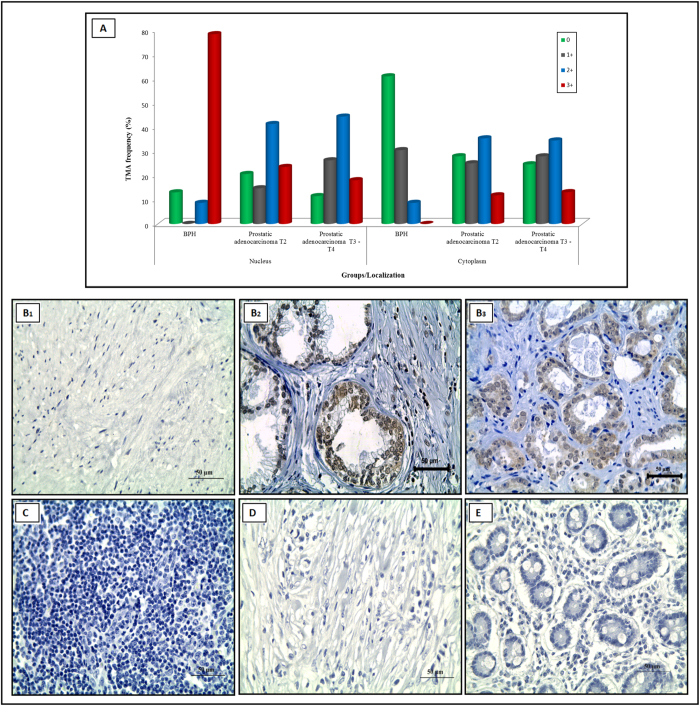
(**A**) Immunoreactivity score (0 to 3) for biotin-labeled CG3-aptamer probe in a tissue microarray (TMA) of BPH and PCa samples. Representative *in situ* hybridization detection of TMA samples (400×). (**B1**) Normal prostate tissue; (**B2**) BPH case with strong nuclear and weak cytoplasmic staining; **(B3)** PCa case with nuclear and moderate cytoplasmic staining; (**C**) Lymphoma; (**D**) Gastric cancer and (**E**) Pancreatic cells.

Representative images of tissues with CG3-aptamer staining are shown in Fig. 4. Biotin-labeled CG3-aptamer was most frequently (78%) localized in the nucleus of BPH cells (Fig. 4A,B2), while it was equally distributed in both cytoplasm and nucleus of PCa cells (Fig. 4B3) and absent in stromal cells.

We observed a homogeneous nuclei-cytoplasm staining of prostatic adenocarcinoma specimens, probably due to a constant *PCA3* RNA production and processing. We were unable to discriminate prostate tumor stages by differential staining intensity.

To determine whether biotin-labeled CG3-aptamer was specific to prostate tissues, we investigated other tumors. In fact, 100% of a lymphoma (Fig. 4C) and gastric cancers (Fig. 4D) tissue specimens stained negatively in both nucleus and cytoplasm. The pancreatic tumor cells (Fig. 4E) presented negative labeling at nuclei, and a very weak cytoplasmic staining (33%) (Table 2).

As previously mentioned, the molecules detected by apta-qPCR consisted of variable transcript sizes (Fig. 3G) suggesting a possible *PCA3* post-transcriptional processing, which may occur inside the nucleus.

Aiming to demonstrate whether CG3-aptamer binds to a specific DNA conformation in the nucleus, we used total non-denatured DNA from PCa and BPH patients as targets for biotin-labeled CG3-aptamer, and performed an apta-qPCR, which resulted in no amplification. Therefore, an explanation for the lack of *PCA3* amplification (qPCR) in both prostate tissues and blood from most BPH patients must still be uncovered.

## Discussion

This is a proof-of-concept study of *PCA3* gene, a long non-coding transcript overexpressed in more than 95% of primary PCa tissue specimens^13^ and the first PCa specific biomarker^14^.

Specific RNA binding is dependent on both sequence and structure of the folded molecule. Our hypothesis about base pair aptamers was that it could be selected against the folded molecule either by regular Watson-Crick associations with non-folded single strands or by interacting with the conformational structure. The immobilization of the single-stranded DNA molecule from a 277-nt fragment, which may have partially paired and bound to the *PCA3* RNA conformational structure, onto magnetic beads was able to expose more accessible “*aptatopes*” for the interaction with the RNA library, and a highly specific aptamer was successfully selected.

Regarding the aptamer selection against ssDNA, we understand that despite the chemical similarity of RNA and DNA backbones, there is evidence from X-ray crystallography that the identity of sugar (ribose *vs.* deoxyribose) affects backbone conformations^23^. Although researchers have used the properties of DNA to understand RNA folding^24^ and previous measurements of flexible single-stranded nucleic acids^25^,^26^,^27^, they have not reached a consensus about conformations. Chen *et al.* demonstrated that ssDNA and ssRNA present differences in conformation while nucleic acids lack secondary structures^28^. However, the same authors have also shown that the effect of the local environment on chain flexibility, in the presence of a flanking double-stranded helix, affects the conformation of single-stranded regions, with implications for biologically relevant nucleic acids. Unfortunately, similar analyses of flexible and disordered regions are still confusing due to the lack of full knowledge on backbone conformations in solution, and how they depend on base content, sugar type, and the presence of salt ions^28^, as have been also shown by different RNA folding softwares (M-fold, S-fold, UNAfold, and others). Due to the stability problems of *PCA3* RNA during the selection process, we have hypothesized that ssDNA could share at least partial conformational structures with ssRNA due to the extensive base-pairing observed inside the *PCA3* molecule. Therefore, we used ssDNA for stability. The enrichment of specific aptamers has later proved that aptamer binds exclusively to *PCA3* mRNA, as shown by specific PCR assays against *PCA3* negative RNA, *PCA3* positive RNA, and DNA.

The identification of trimmed molecules that led to the development of the six aptamers was restricted to the minimal target-binding domain, which has been successfully carried out by alignment. Functional aptamers with less than 40-nucleotides long were obtained, as described elsewhere^29^,^30^,^31^,^32^,^33^,^34^. In general, fixed sequence regions used for primer binding are unimportant for aptamer function and can be eliminated. Advances in aptamer design have been made to eliminate the requirement for the fixed regions in random sequence libraries during the SELEX process, thereby producing short aptamer sequences^35^.

The binding properties of aptamers are due to the formation of specific aptamer/target complexes stabilized by non-covalent interactions. The binding of the aptamer to its cognate target triggers an adaptive folding, in which the target promotes and stabilizes the secondary and tertiary aptamer structures^36^. It can be clearly seen that RNA bases involved in molecular recognition do not form Watson–Crick base pairs, as has been predicted for our CG3-aptamer, demonstrated in Fig. 1. A similar conclusion was reached by Carothers *et al.*^35^, who demonstrated secondary structures for 11 classes of GTP aptamers. Bases with high informational content, which are important for high-affinity binding, are always unpaired and located in loops or bulges. There are two possible reasons for this: first, unpaired RNA bases are more flexible, so they can easily change their conformation to form a binding pocket and accommodate a ligand, and second, unpaired bases have available donor or acceptor atoms for potential formation of hydrogen bonds with the ligand^20^. Therefore, following our aptamer selection criteria, we found that the predicted secondary structure of CG3-aptamer (30 bases) may present at least 26 unpaired bases. It has been suggested that the optimum stability of aptamers is obtained with seven complementary bases^20^, and the greater the number of unpaired bases the lower the possibility to form secondary structures with high free energy. Conversely, the greater the self-folding through aptamer base-pairs the lower their binding affinity. Interestingly, the two most frequent motifs found in our sequences, UGCC and UGUC, were present within the unpaired bases of CG3-aptamer.

Recent studies have shown that aptamers may greatly benefit PCa diagnosis and treatment. At difference from antibodies, aptamers can be generated as molecular beacons or may be conjugated with a variety of functional tags, adapting to many assay formats without losing activity. Thus, taking advantage of their nucleic acid nature, apta-qPCR assays have been developed. Apta-qPCR uses the dual function of aptamers, acting both as a selective ligand to target molecules and as a template for qPCR^2^. Thrombin detection was one of the first targets of an apta-qPCR assay, which has reached as low as a few hundred fM^37^. *E. coli* detection with a mixture of antibody and aptamer followed by qPCR amplification was able to detect ten bacterial cells *per* mL^38^.

Currently, there are only two aptamers developed against proteins involved in PCa. The first and best characterized PCa related aptamer is A10, which binds the prostate-specific membrane antigen (*PSMA*), a tissue marker associated with the beginning and progression of PCa^39^. However, its utility in PCa diagnosis is not clear, and its use has been restricted to *in vitro* inhibition of *PSMA*. The second one is an unmodified RNA aptamer selected against the recombinant prostate-specific antigen (*PSA*) which was able to distinguish between the active and inactive forms of the enzyme^40^, but its diagnostic utility is questionable. Furthermore, *PSA* has severe limitations for PCa detection due to its low specificity and low negative predictive value^41^. This fact has been reinforced by *The United States Preventive Services Task Force* that issued their final recommendation on *PSA* prostate cancer-screening test recommending against routine PSA exams for men of any age^34^.

Currently, the FDA-approved *PCA3* detection in the urine after intense prostatic massage is one of the best available PCa biomarkers with proven utility in the detection and management of early PCa^41^ Similar accuracy has been achieved in peripheral blood^42^, a much less invasive procedure. Therefore, the focus on *PCA3* expression is justified, and aptamers that bind to the native conformation of lnc*PCA3* transcript become a much more interesting target than the simple Watson-Crick base-pairing of either *PSA* or *PSMA* transcripts. This investigation reports the evolution of RNA aptamers against the conformational structure of lncRNA, which could bind to the native folded structure under physiological conditions, without denaturation. An apta-qPCR assay was further developed by a magnetic capture system followed by qPCR amplification and detection of *PCA3* transcripts in blood, which proved to be a very useful tool for PCa diagnosis with excellent sensitivity (100%). We further tested the CG3-aptamer probe in histopathologic assays that also corroborated our findings, evidencing specific and differential staining patterns in PCa and BPH tissue samples.

Aptamers are versatile tools that rival antibodies in diagnostic applications. A classic example of this property is evidenced by an RNA aptamer selected against the constant region (Fc) of the rabbit IgG, which is used as a reporter and may be a secondary marker in several assays where rabbit IgG antibodies are used^43^. Unlike antibodies, synthetic aptamers can easily be produced with a high degree of accuracy, reproducibility and purity. Therefore, little or no batch-to-batch variation is expected in aptamer production. They are not sensitive to temperature and undergo reversible denaturation, thus having a much longer shelf life. Furthermore, the specificity of aptamer recognition may be able to discriminate cognate target molecules and related structures by more than 10.000 to 12.000-fold^44^.

The stronger CG3-aptamer positivity staining in the nuclei of BPH cells suggests that *PCA3* RNA generated in the nucleus might be degraded or have not yet been processed before being exported to the cytoplasm. The diminished *PCA3* expression in the cytoplasm of BPH cells might be due to the loss of these transcripts, and the weaker positive staining may be an indication of the early development of a microfocal tumor event without morphological alterations.

Our histological findings in PCa tissues, demonstrating that *PCA3* is detected in both nucleus and cytoplasm, may explain some of the contrasting results for the subcellular location of *PCA3*, which has been observed at the nucleus^15^,^45^, at microsomal subcellular fractions^15^, and at the cytoplasmic compartment^45^, but not in prostate-tumor stromal cells^15^. We believe that discordances on sensitivity and location of *PCA3* are due to the short splice variants found in both nucleus and cytoplasm, with a predominance of short transcripts at the cytoplasm, which cannot be detected by primers that are not properly designed because of the extensive variation of *PCA3* transcript sequences.

A brief analysis of 88-bp and 140-bp sequences indicated that fragments are only partially transcribed, specifically for exons 1 and 4 (88-bp), and 1, 3 and 4 (140-bp), regions that matched the predicted folded structure of the *PCA3-277* fragment presented in Fig. 1. The only explanation for partial fragments does not rely on an editing process, but in a folded structure that may suffer specific processing and/or cleavage. It is still not known how *PCA3* fragments act by modulating AR signaling and controlling PCa cell survival^15^; however, considering our histological findings of *PCA3* staining in tumor tissues, increased cytoplasmic staining suggests that these fragments might favor tumor occurrence. In addtion, we cannot discard the possibility that such fragments might be precursors of regulatory microRNAs. *PCA3* is clearly absent in normal tissues where transcription is negligible, and the strong staining within the nucleus of BPH tissues may be due to the unprocessed *PCA3*, which is not related to DNA folding because the CG3-aptamer could not bind to the native conformation of DNA molecules. This result raises the possibility that part of the elevated concentration of intranuclear *PCA3* is caused by faulty processing.

In summary, we have demonstrated by *in silico* analysis that the *PCA3* transcript molecule undergoes a significant folding with many hairpins and loops, resulting in a free energy conformational structure. This predicted arrangement might suffer in the nucleus specific post-transcriptional processing during tumor development, exporting smaller folded base-paired fragments to the cytoplasm that may modulate other genes, including the androgen receptor signaling pathway. Such findings were possible due to the development of CG3-aptamer, which was evolved against 277-bases of *PCA3* transcript with high affinity under physiological conditions. This specific aptamer was used in the development of an apta-qPCR assay that captured and amplified *PCA3* transcripts 97-fold and 7-fold higher than conventional qPCR in blood and tissue, respectively. This aptamer was also successfully used for histopathologic analysis through “*in situ* hybridization” using physiological conditions without denaturation with great sensitivity and specificity. Further analysis of apta-qPCR by using agarose gel electrophoresis identified specific and smaller fragments that matched the *PCA3-277* transcript region encompassing parts of the exons 1, 3 and 4, which may be derived from additional RNA processing. Potential novel therapeutic and diagnostic uses of this aptamer are under investigation.

## Patients and Methods/Material

### Aptamer selection

#### Immobilized target molecule

Selection of RNA aptamers with high affinity and specificity to *PCA3* RNA was performed against single-stranded DNA molecule from 277-nt fragment, which may have partially paired and bound to *PCA3* RNA conformational structure, encompassing parts of exons 1, 3 and 4 (alternative splicing of exon 2), which is the most common transcript detected in 95% of PCa tissues^13^.

Briefly, *PCA3*-277 PCR fragment was cloned and purified as previously reported^46^, and biotin was incorporated at the 5′- sense strand of *PCA3*-277 during asymmetric PCR amplification, using 5 pmols of biotinylated forward-primer (5′ biotin - AGATGTTCTTTGATGCGGAGC - 3′), the GeneAmp® dNTPs with dUTP and TaqMan® Universal PCR Master Mix (Applied Biosystems), which, containing AmpErase® UNG, protects against subsequent re-amplification from PCR products containing dUTP to minimize carry-over contamination. UNG incubation at 50 °C was used to cleave any dU‐containing PCR carryover products, and 10 min incubation at 95 °C was used to inactivate the UNG activity, and to denature the native DNA in the experimental sample. PCR assay was performed as previously reported^47^, with minor modifications.

#### Genomic SELEX

For the library construction, we used 1 μg of a pool of genomic DNAs from human PCa tissue specimens (*PCA3*-277 positive). All PCa patients were submitted to radical prostatectomy. The library was generated by random priming of sonicated DNAs, followed by sizing to obtain overlapping fragments of any desired size. The library construction was performed as described elsewhere^5^,^48^.

The *in vitro* transcription of the RNA pool from the library was performed by T7 RiboMAX™, according to manufacturer instructions (Promega). The SELEX was performed using 400 pmols of single-stranded biotinylated *PCA3*-277 (bait uracil) incubated with 10 nmols of estimated 10^14^ RNA aptamers.

PCR products with genome-specific biotinylated primer were batch-purified on streptavidin-coated magnetic beads (Pierce™ - Thermo Scientific). A fresh aliquot of 1 × 10^8^ streptavidin beads was washed 3 times with binding buffer (with 0.02% of Tween 20) before each selection round. The pool of single-stranded biotinylated *PCA3*-277 was heated to 55 °C for 10 min, immediately cooled, and kept at 4 °C for 15 min, followed by a short incubation (5 min) at room temperature before its application in the binding reaction. The *in vitro* selection strategy was designed to identify aptamers that could bind the target under physiological conditions, so aptamers were bound to *PCA3*-277 at 37 °C (pH 7.4) for one hour under gentle shaking. The bead:*PCA3*-277 complex was washed four times with binding buffer to remove non-specific binding. Oligonucleotides complementary to biotinylated single strand were eluted two times by denaturation with NaOH (0.15 M) at 37 °C, for 10 min. The oligonucleotides were precipitated with ethanol, and then resuspended in a smaller volume of binding buffer.

**Figure 5:**
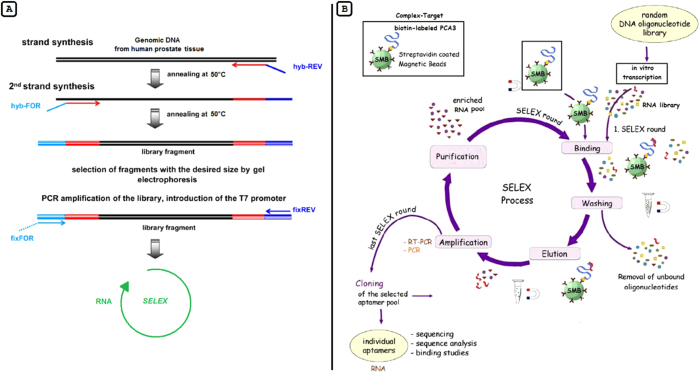
(**A**) Construction of a random DNA oligonucleotide library. Genomic DNA was size-fractionated, size-selected, and PCR amplified to generate the genomic library. Two hybrid primers (hyb) consisting of nine random nucleotides (red) followed by specific sequences (light and dark blue, fix primers) were used to specifically amplify the library. After synthesis, reaction products are size-selected by gel electrophoresis. The fraction that contains library fragments of the desired size range is eluted and amplified by PCR with the fix-primer pair. The T7 promoter (dashed line) for transcription of the library into RNA is incorporated in the fix-FOR primer sequence, according to Zimmermann *et al*.5 with minor modifications. (**B**) Schematic representation of the genomic SELEX, according to Stoltenburg *et al*.1 with modifications. Streptavidin-coated magnetic beads coupled to biotinylated *PCA3* were used to select RNA aptamers, forming a target:aptamer complex. The SELEX procedure consisted of eight cycles of selection steps: binding, washing, elution, amplification and purification. A newly enriched pool of selected oligonucleotides was generated by preparation of the relevant ssDNA for *in vitro* transcription. The enriched aptamer pool was cloned, and several individual aptamers were characterized by sequencing.

During this SELEX process the selected RNA of each round was reverse-transcribed and subsequently amplified by RT-PCR, using fix-REV and fix-FOR primers. The new RNA pool for the next SELEX round was then generated again by *in vitro* transcription (Fig. 5B).

To monitor the enrichment of the selected aptamer pool from SELEX rounds 1, 3 and 8 the sequences were amplified and cloned into pCR2.1-TOPO vector systems (Invitrogen). The purified sequences (192 clones *per* round) were submitted to sequencing (MegaBace 1000), using the DYEnamic Dye Terminator Cycle Sequencing kit (GE Healthcare), and after alignment the sequences were clustered according to repeated motifs. These were identified using the *Clustal software package* of the GCG suite of molecular biology programs. Three sequencing reactions were carried out for each fragment and injected twice to minimize sequencing artifacts.

#### Criteria to evaluate candidate aptamers

The criteria to generate a list of potential RNA aptamers from the 8^th^ selection round consisted of three steps: (1) selection of sequences based on motifs; (2) sequences sizes up to 21 nucleotides long; and (3) significant hybridization energy characteristics between the folded-structure of *PCA3*-277 transcript and selected aptamers (ΔG_hybrid_ ≤ −18.0 kcal/mol). *RNAhybrid*, an extension of the classical RNA secondary structure prediction algorithm^19^, was used for hybridization and nucleic acid folding predictions. The secondary structure analysis was performed by free-energy minimization.

### Apta-qPCR for quantitative detection of *PCA3*-277

#### Ethics statement

This procedure was approved by the UFU Research Ethics Committee (approval number 562.678/2013), along with the Urology Service of the University Hospital. All experiments were performed in accordance with relevant guidelines and regulations.

#### Patients and sample

All patients gave informed consent before specimen collection. Tissue fragments were obtained from 8 PCa patients and frozen in −80 °C before RNA extraction.

Peripheral blood samples were collected before surgery in a vacutainer^™^ tube containing K_2_EDTA 7.2 mg, and maintained at 4 °C. Blood samples from 13 patients were grouped into two classes: 11 with PCa and 2 with benign prostatic hyperplasia (BPH), according to histological classification of tissues. BPH patients were submitted to transurethral resection of prostate (TURP). All PCa patients were submitted to radical prostatectomy. Blood from two young individuals were used as negative controls.

Total RNA extraction from tissue samples was performed as described elsewhere^42^ RNA yield and A260/280 ratio were monitored using a NanoDropND-100 spectrometer (NanoDrop Technologies). For the apta-qPCR approach, 1 μg of total RNA was used from each sample.

#### Apta-qPCR

Synthetic biotinylated anti-*PCA3* aptamers (CG3-aptamer; GenBank/PUIDs: 15262935) were synthesized by Integrated DNA Technology (IDT). Each sample was incubated with 200 pmols of biotin-labeled CG3-aptamer in binding buffer for 30 min, at 37 °C. The biotinylated-CG3:RNA complex was incubated with pre-washed 1 × 10^4^ streptavidin-coated magnetic beads at 37 °C, for 30 min, with mild shaking. Unbound single-stranded RNA sequences were removed by washing three times to remove non-specific binding. The bound RNA was heat-eluted in ultrapure water at 95 °C, for 15 min, and used as template for qPCR amplification.

The amount of RNA *PCA3-277* molecules recovered in each sample was transcribed and quantified by absolute qPCR. To demonstrate the functionality of CG3-aptamer, the results of the apta-qPCR assay was compared with direct qPCR in all samples. Additionally, as positive control, serial dilutions (4 × 10^14^, 2 × 10^14^, 0.9 × 10^14^ and 0.5 × 10^14^ molecules) of single-stranded *PCA3-277* asymmetric PCR fragment (without biotin-labeled) were incubated with biotin-labeled CG3-aptamer, recovered and monitored by qPCR, as previously described above.

#### Construction of standard curve

The 277-bp *PCA3* amplicon was cloned and used to establish the standard curve, as described elsewhere^46^, with serial dilutions of 10^7^, 10^6^, 10^5^, 10^4^, 10^3^ and 10^2^ copies of *PCA3* gene. Linearized plasmid was quantified by spectrophotometry and the number of molecules was estimated according to the formula: copies/μL = [6.023 × 10^23^ × C × OD260 / MW] × 10^−3^, where C = 5 × 10^−5^ g/mL and MW (molecular weight) for *PCA3* = 277 × 658. Absolute quantification was obtained by qPCR carried out in an ABI PRISM 7300 sequence detection system (Applied Biosystems); information on the PCR reaction condition is available on request. The difference between the threshold cycles (Ct) obtained for *PCA3* mRNA (ΔCt) was determined and the results were calculated from the log-linear relationship between the base-10 logarithm of *PCA3* mRNA copy number and ΔCt^49^.

### Tissue microarrays and *in situ* hybridization

Tissue samples were provided by the AC Camargo Hospital Tumor Bank (São Paulo, Brazil). For *in situ* hybridization (ISH), we evaluated a total of 161 cases, including 129 PCa, 23 BPH, 3 lymphomas, 3 gastric cancers, and 3 pancreatic cancers. The median patient age was 63 (46–86) years.

Tissue fragments of 1 mm were obtained from PCa and BPH patients to assemble a tissue microarray (TMA; Beecher Instruments, Sun Prairie/WI) as previously described^50^. Biotin-labeled CG3-aptamer was used to detect *PCA3-277* transcript by ISH analysis.

For ISH reactions, tissue slides were incubated with 1 μg of biotin-labeled CG3-aptamer at 37 °C, for one hour. Slides were washed 3 times with PBS and incubated with horseradish peroxidase (HRP)- streptavidin-conjugated for 5 min. After three more washes using PBS, slides were incubated with the peroxidase substrate, and washed twice using PBS. The slides were counter-stained in Mayer’s hematoxylin for 60 sec, blued with tap water, dehydrated, cleared, and mounted for light microscopy. Endogenous biotin was blocked with avidin-biotin-complex kit (DAKO). Histochemical reactions were performed simultaneously to avoid any bias in the results, due to differences in environmental conditions. The CG3-aptamer probe staining was visually evaluated in both PCa and BPH TMA spots. Percentage of positive cells and staining intensity (scores from 1 to 3) were recorded for each tissue spot. Staining was then categorized as described in Table 3.

### Statistical analysis

*PCA3* expression between two groups was compared using Mann-Whitney’s U-test or the Fisher’s exact test, when appropriate. Results are presented as the mean +/- standard deviation of at least three independent experiments. Probability values below 0.05 were considered statistically significant. Data were analyzed using GraphPad Prism software (San Diego-CA, USA).

## Additional Information

**How to cite this article**: Marangoni, K. *et al.* Prostate-specific RNA aptamer: promising nucleic acid antibody-like cancer detection. *Sci. Rep.* **5**, 12090; doi: 10.1038/srep12090 (2015).

## Supplementary Material

Supplementary Information

## Figures and Tables

**Table 1:** Description of the six best aptamer sequences against *PCA3*.

Aptamer identification	Sequence^a^	Probe unique identifiers	Aptamer size (ntd)	Number of aptamer ntd bound to *PCA3* RNA (ntd in bold)	_Hybrid_ ΔG	Aptamer-*PCA3* loop binding (ntd)	Localization of Aptamer-*PCA3* binding (exon / ntd position)
CD5	AAGGUCUAUUUCCACACCCUU	15262934	21	14	−20.0	9	1 (3 to 22 )
DF7	AGAUGUGGUUCUCCAGCCCCUUGCC	15262937	25	18	−24.0	7	1 (2 to 26 )
BE9	CAUGGUUCCUUCUACAUUUGCCCUAUACCU	15262933	30	10	−18.1	5	1 (3 to 13 )
BC4*	GGUUUAGGUGUGGAUGCCCUUGCU	15262932	24	16	−20.7	10*	3 (186 to 217 )
CG3*	AGUUUUUGCGUGUGUGCCCUUUUUGUCCCC	15262934	30	17	−19.8	12*	3 (194 to 217 )
DB11	UGCGUUCCCAGAUGUGUCCACCGGA	15262936	25	15	−18.5	9	3 (200 to 218 )

^(*)^Aptamers chosen for ISH assay.

^(a)^Motifs were underlined in the sequences.

**Table 2:** Summary of tissue stained with CG3-aptamer probe.

Tissue type	Sample size	Stained nucleus (%)				Stained cytoplasm (%)			
	*Negative*		*Positive*			*Negative*	*Positive*		
		*Weak*	*Moderate*	*Strong*	*Weak*		*Moderate*	*Strong*	
BPH	23	13	–	9	78	61	30	9	–
Prostatic adenocarcinoma T2	68	21	15	41	24	28	25	35	12
Prostatic adenocarcinoma T3 – T4	61	11	26	44	18	25	28	34	13
Lymphoma	3	100	–	–	–	100	–	–	–
Gastric cancer	3	100	–	–	–	100	–	–	–
Pancreatic cancer	3	100	–	–	–	67	33	–	–

**Table 3:** Histopathologic scoring criteria for CG3-aptamer probe staining.

ISH result	Staining intensity	Fraction of stained tumor cells (%)
Negative	*No staining*	
Weak positive*	1+	≥10 <50
	2+	≥10 <30
Moderate positive*	1+	≥50
	2+	≥30 <60
	3+	≥10 <50
Strong positive*	2+	≥60
	3+	≥30

^*^Positive score: ≥10% of stained tumor cells.
