# Supplementary material for: Prostate-specific RNA aptamer: promising nucleic acid antibody-like cancer detection
Source: Sci Rep. 2015 Jul 15;5:12090. doi: 10.1038/srep12090 (PMC4502603; doi:10.1038/srep12090)
Supplement: Supplementary Information [file srep12090-s1.pdf]

Title. Prostate-specific RNA aptamer: promising nucleic acid antibody-like cancer detection

Authors. Karina Marangoni*[1,2], Adriana F. Neves[3], Rafael M. Rocha[4], Paulo R. Faria[5], Patrícia T. Alves[2], Aline G. Souza[2], Patrícia T. Fujimura[2], Fabiana A. A. Santos[2], Thaise G. Araújo[2], Laura S. Ward[1], Luiz R. Goulart[2,6]

Supplementary information

Importantly, other aptamers generated in same selection have been tested by reverse binding assay, but with lower sensitivity and specifically BC4 (Supplementary Table 1) were able to capture only a half of *PCA3* molecules in comparison to CG3.

Approximately 400 pmols of synthetic biotin-labeled aptamer (CG3 and BC4) was incubated with washed 1x10^8 streptavidin-coated magnetic beads at 37°C, for 30 min in a total volume of 200 µL of binding buffer. After binding reaction, unbound aptamers were removed by several washing steps using binding buffer.

The reverse binding assay was performed in three different conditions: complex – streptavidin-coated magnetic beads/biotin-labeled aptamer – was resuspended in 500 µL binding buffer containing (I) 10 nmol of *PCA3*, (II) 10 nmols of *PCA3* pre-incubated with specific-aptamer without biotin-labeled at 37°C, for one hour with mild shaking and (III) 10 nmol of scramble non-specific RNA. In all experiments were used *PCA3* RNA without biotin-labeled. Incubation, washing and elution steps were performed as reported above. The amount of *PCA3* recovered in each approach is an indicator of affinity and specificity of selected RNA aptamer binds *PCA3* (Supplementary Table 1).

Supplementary Table 1. Reverse binding assay performance

**Aptamers**	***PCA3* recovered (RQ)** *Conditions*		
	**I**[a]	**II**[b]	**III**[c]
**CG3**	2.5512,27	235,12	*insignificant*
**BC4**	1.111,35	108,02	*insignificant*

(a) Complex incubated with purified *PCA3*; **(b) C**omplex incubated with *PCA3* pre-incubated with aptamer without biotin-labeled**; (c)** Complex incubated with scramble non-specific RNA.
